# Isolation, plant colonization potential, and phenanthrene degradation performance of the endophytic bacterium *Pseudomonas* sp. Ph6-*gfp*

**DOI:** 10.1038/srep05462

**Published:** 2014-06-26

**Authors:** Kai Sun, Juan Liu, Yanzheng Gao, Li Jin, Yujun Gu, Wanqing Wang

**Affiliations:** 1 Institute of Organic Contaminant Control and Soil Remediation, College of Resources and Environmental Sciences, Nanjing Agricultural University, Nanjing 210095, China; 2 These authors contributed equally to this work.

## Abstract

This investigation provides a novel method of endophyte-aided removal of polycyclic aromatic hydrocarbons (PAHs) from plant bodies. A phenanthrene-degrading endophytic bacterium *Pseudomonas* sp. Ph6 was isolated from clover (*Trifolium pratense* L.) grown in a PAH-contaminated site. After being marked with the GFP gene, the colonization and distribution of strain Ph6-*gfp* was directly visualized in plant roots, stems, and leaves for the first time. After ryegrass (*Lolium multiflorum* Lam.) roots inoculation, strain Ph6-*gfp* actively and internally colonized plant roots and transferred vertically to the shoots. Ph6-*gfp* had a natural capacity to cope with phenanthrene *in vitro* and *in planta*. Ph6-*gfp* degraded 81.1% of phenanthrene (50 mg·L^−1^) in a culture solution within 15 days. The inoculation of plants with Ph6-*gfp* reduced the risks associated with plant phenanthrene contamination based on observations of decreased concentration, accumulation, and translocation factors of phenanthrene in ryegrass. Our results will have important ramifications in the assessment of the environmental risks of PAHs and in finding ways to circumvent plant PAH contamination.

Polycyclic aromatic hydrocarbons (PAHs) are a group of highly toxic and recalcitrant organic contaminants that are widely distributed in environments throughout the world^1^,^2^. Large amounts of PAHs and their toxic metabolites caused by incomplete biodegradation that are present in soils can be taken up by plant roots, accumulated, and translocated into plant shoots. This uptake is the major route of PAH entry into the food chain/web, resulting in serious threats to human health and the ecosystem^2^,^3^,^4^. Obtaining the knowledge and ability to reduce the uptake of PAHs by plants will have considerable benefits in ecological risk assessments^4^,^5^,^6^.

Plant-associated microorganisms play key roles in PAH uptake by plants^7^,^8^. These microbes can participate in PAH-degrading processes^9^. The inoculation of arbuscular mycorrhizal fungi (AMF) has proven to be effective in reducing the PAH concentrations in soils^10^. Recently, a study further reported that AMF hyphae could mediate the uptake of PAHs by ryegrass roots. In a three-compartment system, in which plant roots were grown in uncontaminated soils, the hyphae of *Glomus mosseae* and *Glomus etunicatum* extended from the root surface into PAH-contaminated soil to absorb PAH and transport them to the plant roots, resulting in high PAH concentrations in the roots^11^. PAH-degrading bacteria have also been isolated and utilized in the degradation of PAHs. The functional bacterial strain PS4040 (*Leclercia adecarboxylata*), isolated from oily sludge-contaminated soil, was able to degrade pyrene by using it as its sole carbon source^12^. The rhizobacterium *Sinorhizobium meliloti* P221 could colonize the rhizosphere soil and enhance phenanthrene degradation by producing surfactants and indole-3-acetic acid (IAA, capable of promoting plant growth)^13^. However, the reported PAH-degrading bacteria have been overwhelmingly isolated from soil, sediment, and sludge, and little information is available on the enormous potential of endophytes in mediating the uptake of PAHs by plants^14^.

Endophytes reside in the internal tissues of healthy or symptomless plants without causing apparent harm to their hosts^15^. They are ubiquitous in different vegetative parts of plants, such as the roots, tubers, stems, and leaves^16^,^17^,^18^. Certain endophytes benefit plant growth, increase plant tolerance, and promote degradative capacity^19^,^20^,^21^. For example, several endophytic bacteria isolated from hybrid poplar trees had the ability to tolerate a range of target pollutants and to improve the ability to degrade BTEX-compounds (benzene, toluene, ethylbenzene, and xylene) in soil^22^. Moreover, endophytes can also colonize various plant species, maintain stable relationships with plants, and improve biodegradation capabilities of organic contaminants^23^. A pyrene-degrading endophytic bacterium *Enterobacter* sp. 12J1, isolated from *Allium macrostemon* Bunge, could colonize both wheat and maize and promote pyrene removal from contaminated soil^8^. Unfortunately, only a very small number of PAH-degrading endophytes have been isolated thus far, and most research has concerned their utility in PAH biodegradation in soil environments. Little literature is available on the utility of PAH-degrading endophytes in reducing PAH contamination in plants. In addition, the ability to track the colonization and distribution of inoculated endophytes within host plants is of great concern.

The green fluorescent protein (GFP) gene marker has been widely used to visualize and track the colonization patterns of bacterial strains within inoculated host plants^24^,^25^,^26^. It provides a unique and visual phenotype for studying the population dynamics of microorganisms within plant tissues. Germaine *et al*.^27^ found that after a period of inoculation of poplar trees, several *gfp*-labeled poplar endophytes were detected in all of the interior tissues of the poplar trees. Ferreira *et al*.^28^ documented that the endophytic bacterium *Pantoea agglomerans* 33.1, tagged with the GFP gene, was detected to have colonized *Eucalyptus* roots, mainly in intercellular spaces, stems, and xylem vessels. However, to date, few documents are available on the utility of the GFP gene marker in tracking the colonization and distribution of PAH-degrading endophytes in host plants^21^,^29^.

In this paper, the phenanthrene-degrading endophytic bacterium *Pseudomonas* sp. Ph6 was isolated from clover (*Trifolium pratense* L.) grown in PAH-contaminated soil. After being *gfp*-tagged, Ph6-*gfp* colonization and its performance on PAH uptake by ryegrass (*Lolium multiflorum* Lam.) were systematically investigated. Large areas contaminated with PAHs can be found in many countries. The ability to reuse these fields and produce safe agricultural products is of great interest. Our study provides a novel method of endophyte-aided removal of PAHs from plant bodies, which can benefit agricultural production, food security, and human health in areas contaminated by organic pollutants.

## Results

### Isolation, identification, and *gfp*-labeling of *Pseudomonas* sp. Ph6

**Figure 1:**
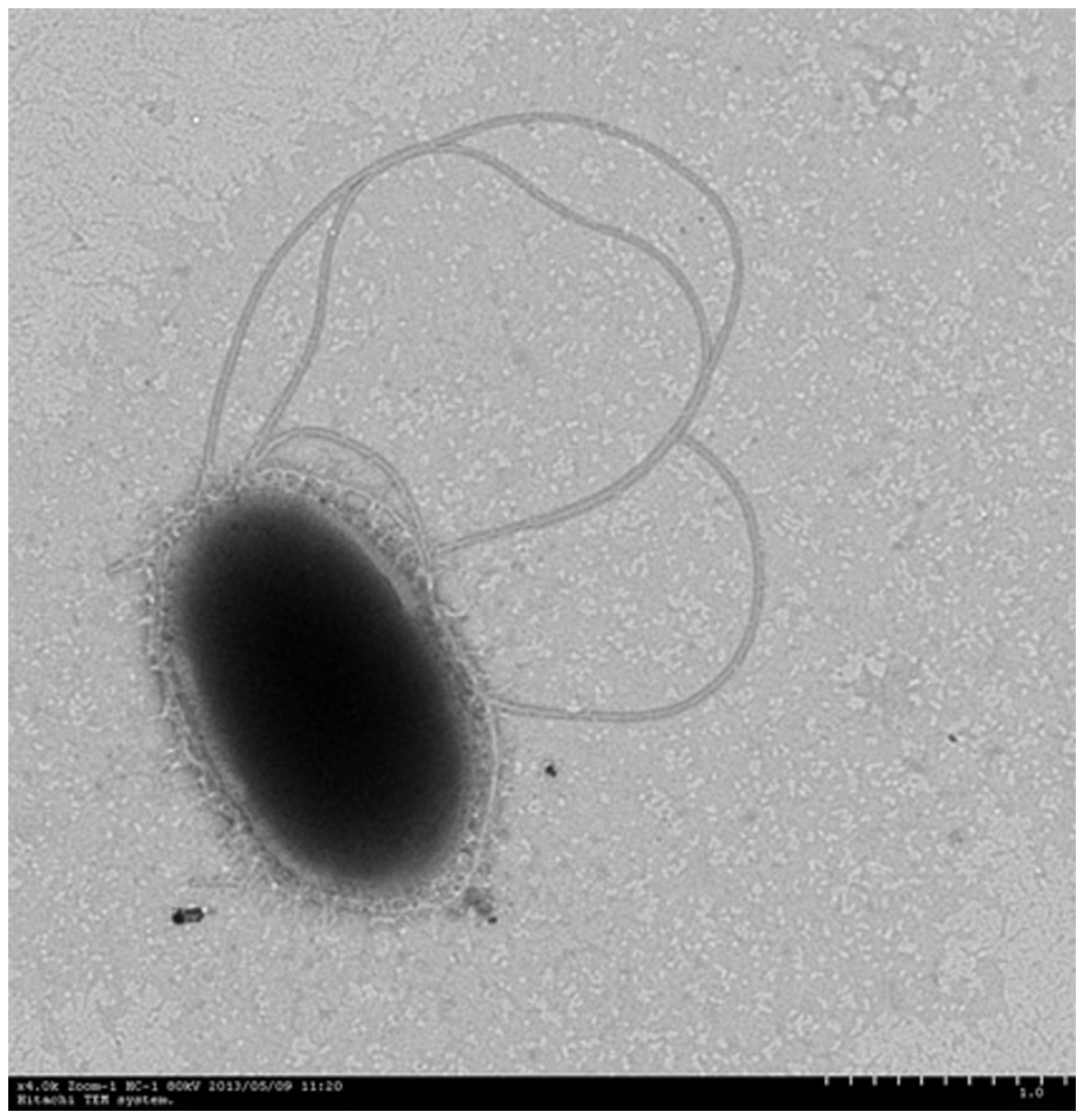
Transmission electron micrograph of strain Ph6 isolated from clover (*Trifolium pratense* L.) (×4.0 K Zoom-1 HC-1 80 kV).

The phenanthrene-degrading endophytic bacterium *Pseudomonas* sp. Ph6 was isolated from the interior of clover plants (*Trifolium pratense* L.) grown in a PAH-contaminated site near a petrochemical plant. This strain is a rod-shaped, aerobic, gram-negative bacterium that is motile, with polar flagella. Ph6 was positive for nitrate reductase, negative for indole and gelatin liquefaction, and positive for malate, citric acid, and phenylacetic acid. The glucose test was positive, while mannose and maltose were negative. The 16S rRNA gene sequence of strain Ph6 showed 99% identity to *Pseudomonas* sp. The GenBank accession number of the 16S rRNA gene sequence of strain Ph6 is KF741207. On the basis of its morphology, physiology, and 16S rRNA gene sequence analysis, Ph6 was identified as a *Pseudomonas* sp. bacterium. The cellular morphology of strain Ph6 is shown in Fig. 1.

**Figure 2:**
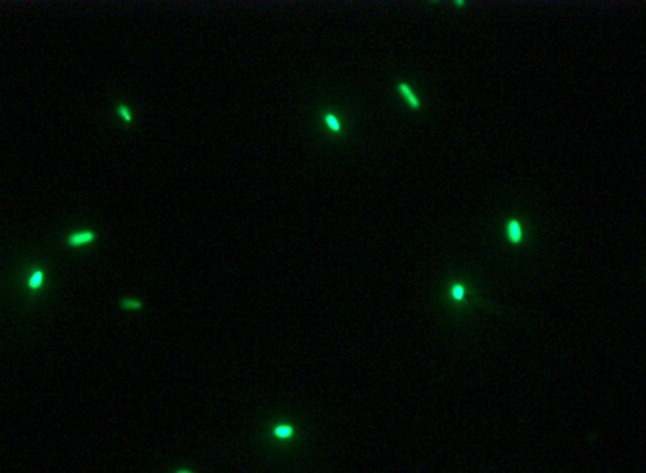
Fluorescence of strain Ph6-*gfp* in culture solution (×100). The wild endophytic bacterium *Pseudomonas* sp. Ph6 tagged with *gfp* (Ph6-*gfp*) was observed under fluorescence microscopy. Bacterial cells with green fluorescence were displayed as bright green rods on a glass microscope slide.

**Figure 3:**
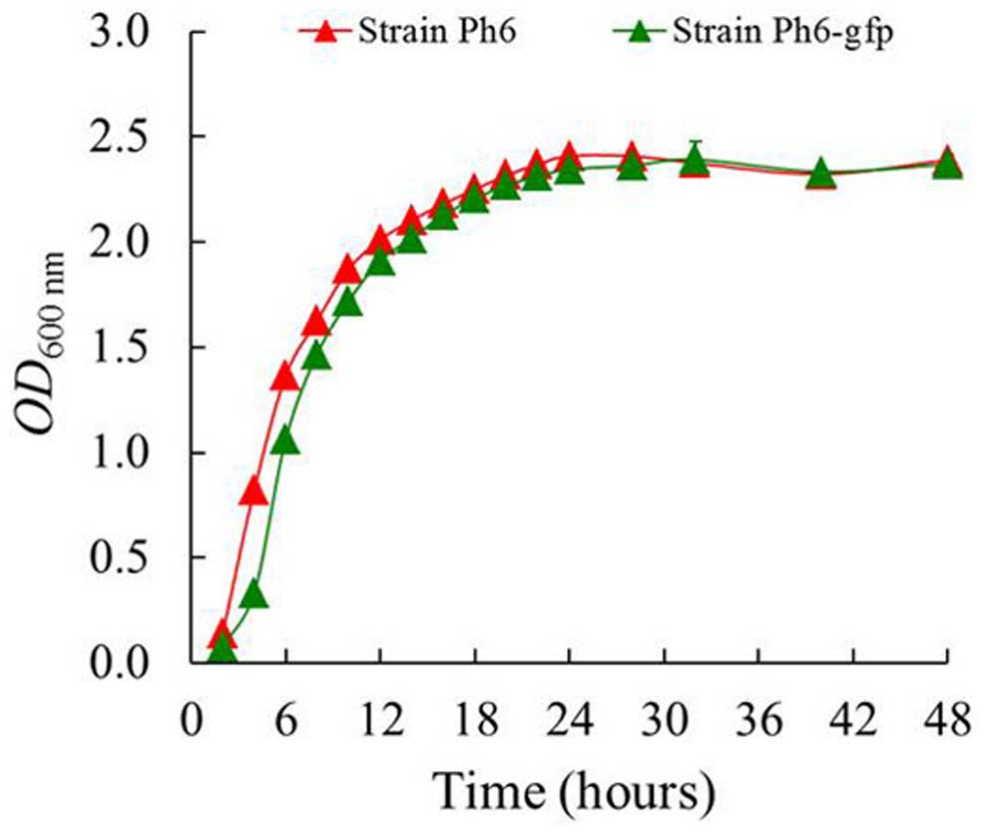
Growth curves of strains Ph6 and Ph6-*gfp*. The growth of wild endophytic bacterium *Pseudomonas* sp. Ph6 (Red line) and strain Ph6 tagged with *gfp* (Green line) in Luria-Bertani (LB) medium (pH 7.0) for 48 hours. Error bars are standard deviations (n = 3); in some case, these are not visible as they are smaller than the graph points.

The plasmid pBBRGFP-45 was successfully transformed into endophytic bacterium *Pseudomonas* sp. Ph6, and strain Ph6 tagged with GFP gene (Ph6-*gfp*) showed stable *gfp* activity. The presence of the plasmid pBBRGFP-45 in the transconjugants was confirmed by PCR (data not shown). The cells carrying the plasmid were detected by fluorescence microscopy (Fig. 2). The morphologically tagged strain *Pseudomonas* sp. Ph6-*gfp* was highly similar to the wild strain *Pseudomonas* sp. Ph6, and the growth rate of Ph6-*gfp* in liquid LB was also similar to the wild type (Fig. 3). This result indicates that the presence of the plasmid did not interfere with the normal growth and activity of Ph6.

### Biodegradation of phenanthrene by Ph6-*gfp* in culture solution

**Figure 4:**
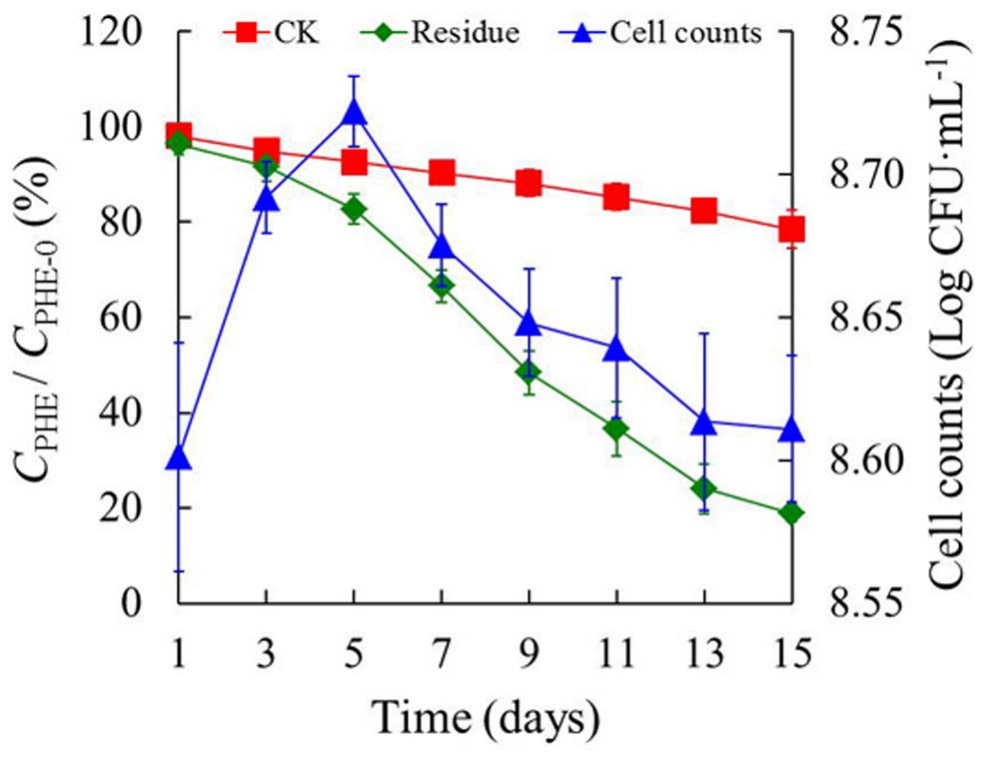
The degradation kinetics of phenanthrene and growth curves of strain Ph6-*gfp* in culture solution. *C*_PHE_ represents the residual concentration of phenanthrene in solution at a given time. *C*_PHE-0_ represents the initial concentration of phenanthrene in solution. After the inoculation of strain Ph6-*gfp*-free (Red line) and strain Ph6-*gfp* (Green line) in a high PAH-contaminated culture solution (50 mg·L^−1^ Phenanthrene) for 15 days, the residual rate of phenanthrene was detected by high-performance liquid chromatography (HPLC), and the growth of strain Ph6-*gfp* (Blue line) was detected by counting the CFU on plates. Error bars are standard deviations (n = 3); in some case, these are not visible as they are smaller than the graph points.

Strain Ph6-*gfp* had phenanthrene degradation efficiency similar to that of the wild strain Ph6, which could degrade 85% of phenanthrene in a culture solution within 15 days. The degradation kinetics of phenanthrene and growth curves of strain Ph6-*gfp* were studied in the same culture solution as the wild strain (Fig. 4). The results indicate that strain Ph6-*gfp* grew well and degraded phenanthrene effectively in 15 days. Cell counts of Ph6-*gfp* first rose and then fell after 15 days. The initial and maximum values (At day 5) of the cell counts were 8.60 and 8.72 (Log CFU·mL^–1^), respectively. The concentration of phenanthrene in the culture solution with Ph6-*gfp* decreased consistently with time, and 81.1% of the phenanthrene was dissipated in 15 days. In the control solution (Ph6-*gfp*-free), only 21.6% of the phenanthrene disappeared due to abiotic dissipation in 15 days. This result indicates that Ph6-*gfp* was effective in phenanthrene degradation in culture medium. The data above show that the GFP gene did not disrupt any key traits required for survival or phenanthrene degradation of strain Ph6 and that Ph6-*gfp* could, in principle, be used to degrade phenanthrene in inner plant tissues.

### Colonization and distribution of Ph6-*gfp* in plants

**Figure 5:**
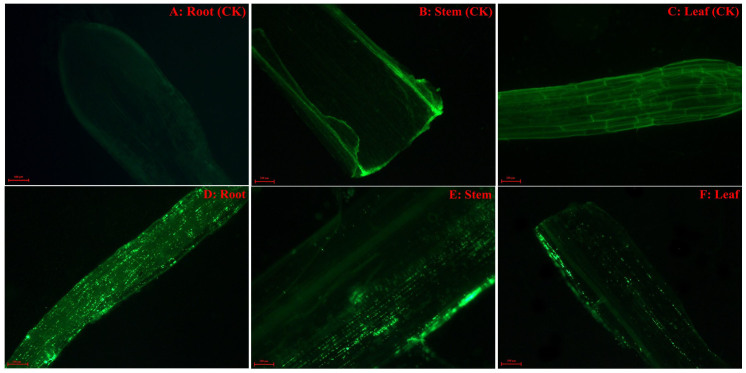
Visualization of inoculated endophyte Ph6-*gfp* within plant tissues. After inoculation with Ph6-*gfp* for 6 days, the colonization of Ph6-*gfp* was observed in plant roots, stems and leaves by fluorescence microscopy. Bacterial cells with green fluorescence were displayed as bright green rods inside roots (D), stems (E) and leaves (F). Plants with Ph6-*gfp*-free inoculation served as the control (A, B, C).

Endophytic strain Ph6-*gfp* was an efficient colonizer of phenanthrene-contaminated ryegrass and could vertically transfer from roots to shoots. When ryegrass roots were soaked in a suspension of Ph6-*gfp* for 6 hours, Ph6-*gfp* first colonized the plant root surface and formed bacterial aggregates or biofilms. Strain Ph6-*gfp* then proceeded to enter into the plant roots (Fig. 5D). As time progressed, Ph6-*gfp* cells were also visualized in the stems (Fig. 5E) and leaves (Fig. 5F). Plants with Ph6-*gfp*-free inoculation served as the control (Fig. 5A–C). The quantification of Ph6-*gfp* within plant roots and shoots was performed by counting the CFU on plates (Table 1). The cell counts of Ph6-*gfp* decreased progressively from ryegrass roots to stems to leaves, and cell counts in the roots were significantly (p < 0.01) higher than those in the shoots. The localization and distribution of strain Ph6-*gfp* within the plants changed with time. The cell counts of Ph6-*gfp* within the plants initially increased for the first 6 days and then gradually decreased from days 6–15. After inoculation for 15 days, the cell counts of Ph6-*gfp* were 5.51 and 3.65 log CFU·g^−1^ in roots and shoots, respectively. In addition, strain Ph6-*gfp* could also be re-isolated and released into Hoagland solution, and cell counts were observed between 10^4^ and 10^6^ CFU·mL^−1^ in solution. The colonization of Ph6-*gfp* provided the premise for the utilization of this endophytic strain to influence phenanthrene uptake by plants.

### Performances of endophyte mediate the uptake of phenanthrene by plants

Phenanthrene-degrading endophytic bacterium Ph6-*gfp* could significantly reduce the risks associated with plant PAH-contamination based on observations of decreased concentration and accumulation of phenanthrene in plant bodies. Furthermore, the translocation of phenanthrene from roots to shoots decreased in endophyte-colonized plants.

**Figure 6:**
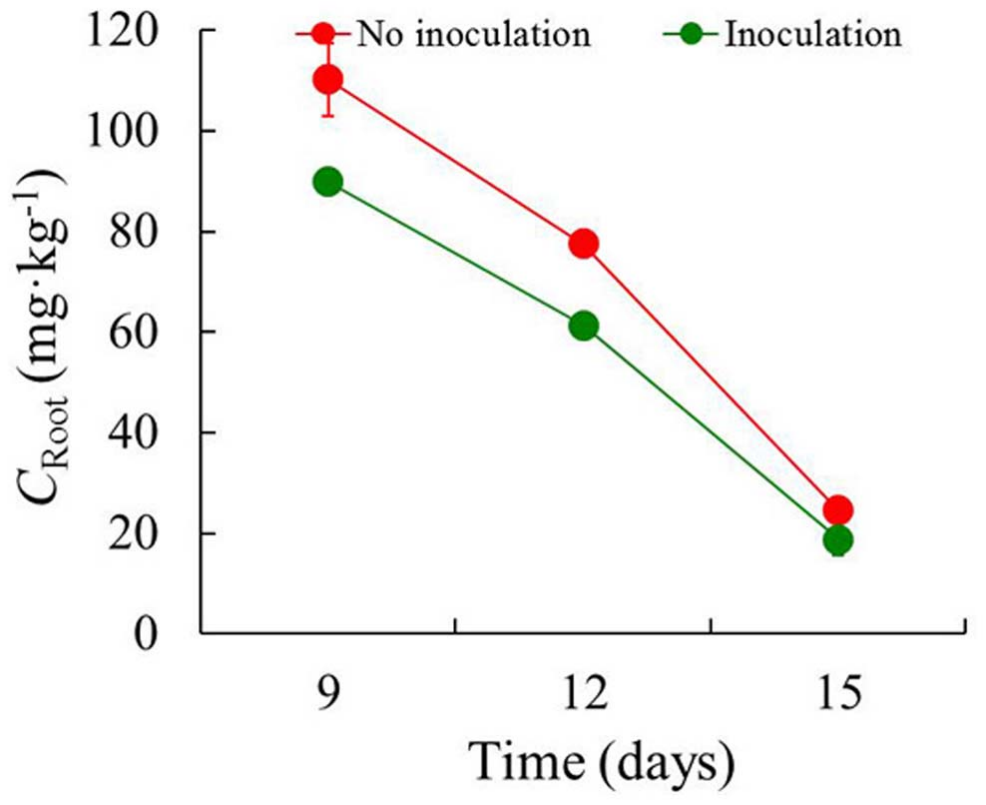
The concentration of phenanthrene in plant roots as a function of time. *C*_Root_ represent the concentration of phenanthrene in plant roots. After Ph6-*gfp* inoculation for 9, 12 and 15 days in PAH-contaminated plants, the concentrations of phenanthrene in Ph6-*gfp*-colonized (Green line) and Ph6-*gfp*-free (Red line) roots were detected by HPLC. Error bars are standard deviations (n = 3); in some case, these are not visible as they are smaller than the graph points.

**Figure 7:**
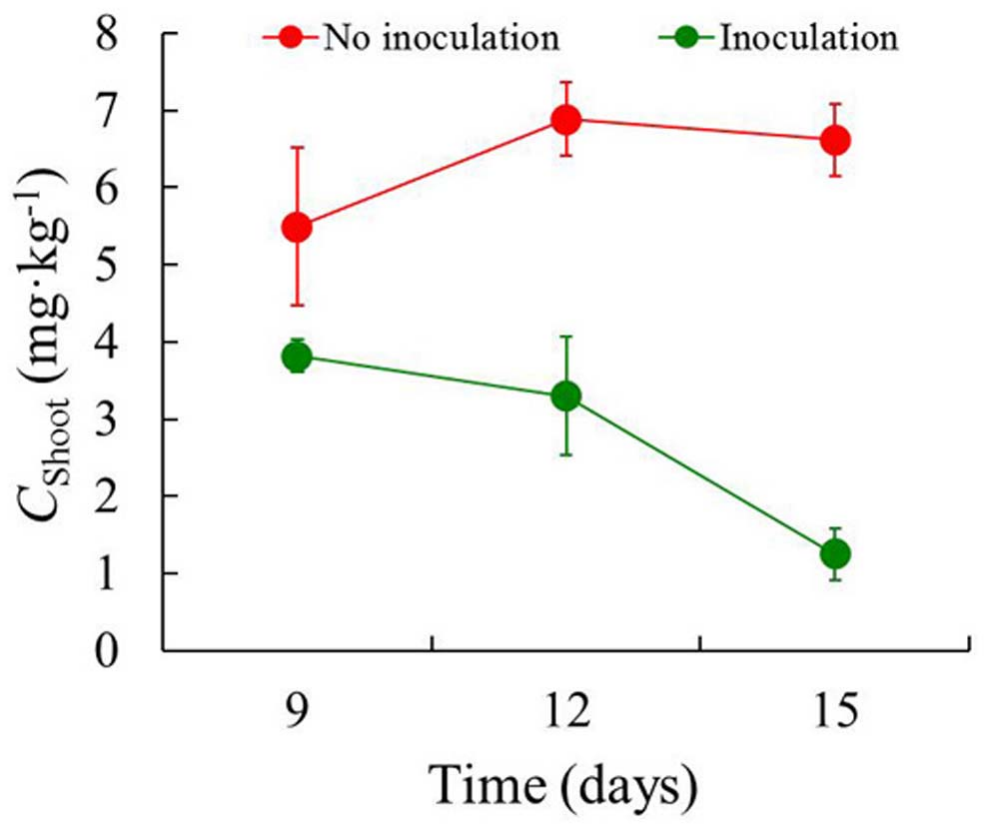
The concentration of phenanthrene in plant shoots as a function of time. *C*_Shoot_ represent the concentration of phenanthrene in plant shoots. After Ph6-*gfp* inoculation for 9, 12 and 15 days in PAH-contaminated plants, the concentrations of phenanthrene in Ph6-*gfp*-colonized (Green line) and Ph6-*gfp*-free (Red line) shoots were detected by HPLC. Error bars are standard deviations (n = 3); in some case, these are not visible as they are smaller than the graph points.

Strain Ph6-*gfp* showed a natural capacity to cope with phenanthrene *in vitro* as well as within plants (Fig. 4, 6 and 7). The concentrations of phenanthrene in ryegrass roots and shoots with and without Ph6-*gfp* inoculation are shown in Fig. 6 and 7. After Ph6-*gfp* inoculation for 9–15 days, the concentrations of phenanthrene in endophyte-colonized roots and shoots were 18.8–89.8 and 1.25–3.82 mg·kg^−1^, respectively, whereas the concentrations in roots and shoots colonized by the Ph6-*gfp*-free inoculation were 24.5–110 and 5.49–6.89 mg·kg^−1^, respectively. The concentrations of phenanthrene in endophyte-colonized roots and shoots decreased in 9–15 days. Compared with the Ph6-*gfp*-free treatment, the respective concentrations of phenanthrene in endophyte-colonized roots and shoots were 18.5%–23.3% and 30.4%–81.1% lower, respectively. With the inoculation with Ph6-*gfp*, a more significant decrease in phenanthrene concentration was observed in the shoots than in the roots. These results indicate that Ph6-*gfp* aided phenanthrene degradation in contaminated ryegrass and significantly reduced plant phenanthrene contamination. In addition, the concentrations of phenanthrene in roots were always one to two magnitudes larger than those in shoots, irrespective of the inoculation with Ph6-*gfp*.

The inoculation of ryegrass with strain Ph6-*gfp* can also reduce the accumulated amounts (*A*, μg·bottle^−1^) of phenanthrene in plants. *A* was estimated according to the following equation: 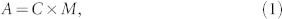
where *C* (mg·kg^−1^) is the concentration of phenanthrene in the root or shoot, and *M* (g·bottle^−1^, on a dry weight basis) is the biomass of ryegrass root or shoot in each bottle. Higher *A* values indicate more phenanthrene present in the plants and higher risk of plant contamination. The calculated *A* values are given in Table 2. The accumulation of phenanthrene in ryegrass shoots was much smaller than in roots, irrespective of Ph6-*gfp* inoculation; i.e., the root was the dominant sink of the phenanthrene present in the plant bodies, although shoots were also extensively contaminated by phenanthrene. After 9–15 days, the accumulated amounts of phenanthrene in endophyte-colonized roots and shoots were 0.87–2.30 and 0.17–0.31 μg·bottle^−1^, respectively, corresponding to values 10.3%–17.6% and 30.8%–66.5% lower than those in Ph6-*gfp*-free plant roots and shoots. This result indicates that the inoculation of ryegrass with Ph6-*gfp* reduced phenanthrene accumulation in plant bodies. Moreover, similar to the trend of plant phenanthrene concentrations, inoculation with Ph6-*gfp* produced a decrease in phenanthrene accumulation that was more significant in the ryegrass shoots than in the roots. In addition, compared with those at day 12, the *A* values in roots and shoots were smaller at day 15, irrespective of the inoculation with Ph6-*gfp*, suggesting the obvious metabolism of phenanthrene in plants.

**Figure 8:**
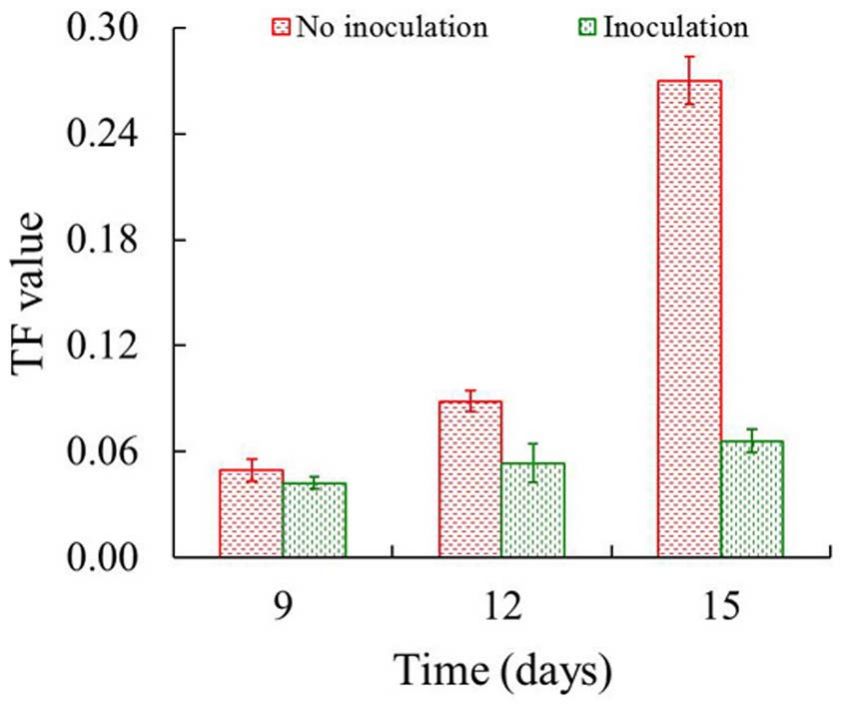
The translocation factor (TF) of phenanthrene in ryegrass as a function of uptake time. TF = *C*_Shoot_/*C*_Root_, where *C*_Shoot_ and *C*_Root_ are the phenanthrene concentrations in plant shoots and plant roots, respectively. A larger TF value infers a more significant translocation of phenanthrene from root to shoot. Red and green bars indicate the inoculation of strain Ph6-*gfp*-free and Ph6-*gfp*, respectively. Error bars are standard deviations (n = 3).

The translocation factor (TF, unitless) of phenanthrene in ryegrass was estimated as 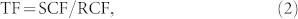
where RCF and SCF are the root and shoot concentration factors, respectively. RCF and SCF were obtained according to the following equations: 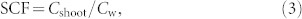


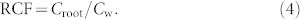
Therefore, by combining Equations 2–4, TF could be further estimated as 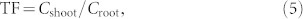
where *C*_shoot_, *C*_root_, and *C*_w_ are the phenanthrene concentrations in plant shoots, plant roots, and Hoagland solution, respectively. A larger TF value infers a more significant translocation of phenanthrene from root to shoot. As shown in Fig. 8, the TF values increased from days 9 to 15, irrespective of Ph6-*gfp* inoculation. The TF values of phenanthrene in Ph6-*gfp*-free ryegrass in days 9–15 were 0.05–0.27, which were 25.0%–286% greater than that in Ph6-*gfp*-inoculated plants. These results indicate that strain Ph6-*gfp* efficiently impeded phenanthrene translocation from the roots to the shoots of ryegrass.

## Discussion

PAHs can actually be absorbed by plants, transported from roots to shoots, following the transpiration stream, and be partially metabolized by plants^30^,^31^. However, large quantities of PAH residue remain in plants, posing serious threats to food security and human health. As such, the capability for the reduction of the PAHs residue in plants is of worldwide concern. Because PAHs are pollutants with highly toxic properties, even small enhancements in the removal of PAHs from plants would be of great importance. Our study was a primary investigation on the utilization of endophytes to enhance PAH degradation in plants.

Endophytes can not only abundantly reside in the internal tissues of plants but also have rich variety and different functions^15^,^19^,^20^,^21^. Until now, several endophytic bacterial species were isolated from plants that grew well in the presence of naphthalene, catechol, and phenol, and it was discovered that these strains could utilize the pollutants as their sole carbon source and energy source for growth^32^,^33^. A phenanthrene-degrading endophytic bacterium strain Ph6 was isolated from the interiors of PAH-contaminated clover plants, indicating that PAH-degrading endophytes can reside in the internal tissues of PAH-contaminated plants and may have specialized functions in plants^29^. Moreover, our result will also potentially increase the resources of PAH-degrading bacterial species and enrich the pool of PAH-degrading genes^21^.

*Pseudomonas* sp. can be beneficial in the protection of plants from soils contaminated by organic pollutants^21^,^34^,^35^,^36^. The poplar endophyte *Pseudomonas putida* VM1441 was an efficient colonizer of interior plant root tissues and allowed the host plant to circumvent the phytotoxic effects of naphthalene^35^. Furthermore, with the colonization of the endophytic bacterium *Pseudomonas putida* 1450, the pea plant *Pisum sativum* displayed no 2,4-dichlorophenoxyacetic acid (2,4-D) accumulation in plant aerial tissues^36^. However, few previous investigations have tracked the visualization to elucidate the performance of PAH-degrading functional endophytes in plants. We demonstrated that the PAH-degrading endophytic bacterium *Pseudomonas* sp. Ph6 tagged with the GFP gene can re-colonize PAH-contaminated plants and be transferred from roots to stems and leaves. Furthermore, Ph6-*gfp* has a natural capacity to cope with phenanthrene *in planta* (Fig. 6 and 7), which provides the possibility of utilizing endophyte Ph6-*gfp* to degrade PAHs in plants.

Large quantities of PAH-degrading endophytes colonize plant interiors and play a significant role by promoting the degradation of PAHs in plants. The endophytic populations observed in spruce were between 2.0 and 7.0 log CFU·g^−1^ FW^37^, and the colonies of *Pseudomonas* sp. cells in the interior root and shoot tissues of inoculated poplar trees were between 2.0 and 5.0 log CFU·g^−1^ FW^27^. In our study, after the ryegrass roots were soaked with the suspension of strain Ph6-*gfp*, the highest densities of strain Ph6-*gfp* were observed in the roots (5.51–5.79 log CFU·g^−1^ FW), and densities decayed progressively from the stems to the leaves. This result was similar to that observed for *Herbaspirillum seropedicae* sp. in rice seedlings, even though the concentration of *H. seropedicae* sp. cells was much higher (6 log CFU·g^−1^ FW)^38^. This indicates that PAH-degrading endophytes can readily adapt to inner plant circumstances and aid in the elimination of the risks involved with plant PAH contamination.

The possible mechanisms involved in the endophyte-enhanced degradation of PAHs in plants include the following: (1) Endophytes can be released into soil and promote PAHs degradation in the environment and thereby reduce the uptake of PAHs by plant roots^39^; (2) Endophytes not only degrade PAHs *in vitro* but can have the same effect *in planta*^39^; (3) Endophytic inoculants can transfer their PAH-degradative genes to other endophytes in plants, thus increasing the overall degradation potential of endogenous endophytic communities^40^,^41^; (4) Endophytes may influence the activities of plant enzymes, such as oxidase, reductase, esterase, and dehalogenase, participating in the transformation of PAH contaminants^29^,^42^,^43^; (5) Endophytes may promote PAH-metabolizing gene expression, such as alkane monooxygenase (*alk*B) and naphthalene dioxygenase (*ndo*B)^44^.

Ryegrass is known to be a common forage grass, and the shoots are an important food for livestock. Therefore, the ability to reuse PAH-contaminated fields and produce safe ryegrass plants is of great interest. In greenhouse container experiments, with the inoculation of endophyte Ph6-*gfp* in artificially contaminated-soil (Phenanthrene: 1 mg·kg^−1^), the contents of phenanthrene in ryegrass plants decreased markedly. After Ph6-*gfp* inoculation for 15 days, the concentrations and accumulated amounts of phenanthrene in endophyte-colonized roots and shoots were 50.19 ± 0.84, 11.25 ± 2.40 mg·kg^−1^ and 1.31 ± 0.11 and 0.52 ± 0.02 μg·pot^−1^, respectively, corresponding to values 7.9%, 27.3% and 6.4%, 23.2% lower than those in Ph6-*gfp*-free plant roots and shoots. Notably, endophyte Ph6-*gfp* inoculation reduced the risk of PAH contamination in shoots to a greater degree than that in roots. Clearly, the research revealed that endophytic bacteria, and possibly fungi, could be exploited to address plant PAHs contamination and allow the food chain/web to circumvent these chemicals. Nevertheless, how to enhance the implication of endophytes in PAH-contaminated fields is of great significance.

## Methods

### Chemicals and media

Phenanthrene (PHE), as a representative PAH, was purchased from Fluka (Neu-Ulm, Germany) with a purity exceeding 98%. This PAH was selected because it is commonly found in contaminated soils and has been examined in many environmental studies^11^. The molecular weight (*M*_w_), water solubility (*S*_w_), and log-transformed octanol-water partition coefficient (Log *K*_ow_) are 178.23 g·mol^−1^, 1.18 mg·L^−1^, and 4.57, respectively^45^.

The mineral salt medium (MSM) contained 1.50 g·L^−1^ (NH_4_)_2_SO_4_, 1.91 g·L^−1^ K_2_HPO_4_·3H_2_O, 0.50 g·L^−1^ KH_2_PO_4_, 0.20 g·L^−1^ MgSO_4_·7H_2_O, and 1 mL of trace element solution (0.1 mg·L^−1^ CoCl_2_·6H_2_O, 0.425 mg·L^−1^ MnCl_2_·4H_2_O, 0.05 mg·L^−1^ ZnCl_2_, 0.01 mg·L^−1^ NiCl_2_·6H_2_O, 0.015 mg·L^−1^ CuSO_4_·5H_2_O, 0.01 mg·L^−1^ Na_2_MoO_4_·2H_2_O, 0.01 mg·L^−1^ Na_2_SeO_4_·2H_2_O). Phenanthrene (50 mg·L^−1^) was added to MSM to make a PMM medium. Luria-Bertani (LB) medium (pH 7.0) contained 10.0 g·L^−1^ tryptone, 5.0 g·L^−1^ yeast extract, and 10.0 g·L^−1^ NaCl.

Hoagland nutrient solution (pH 5.5) consisted of 945 mg·L^−1^ Ca(NO_3_)_2_·4H_2_O, 506 mg·L^−1^ KNO_3_, 80 mg·L^−1^ NH_4_NO_3_, 136 mg·L^−1^ KH_2_PO_4_, 493 mg·L^−1^ MgSO_4_, iron salt solution (2.5 mL): 2.78 g FeSO_4_·7H_2_O, 3.73 g EDTA·Na_2_, and 500 mL of distilled water. Trace element solution (5 mL, pH 6.0) contained 0.83 mg·L^−1^ KI, 6.2 mg·L^−1^ H_3_BO_3_, 22.3 mg·L^−1^ MnSO_4_, 8.6 mg·L^−1^ ZnSO_4_, 0.25 mg·L^−1^ Na_2_MoO_4_, 0.025 mg·L^−1^ CuSO_4_, and 0.025 mg·L^−1^ CoCl_2_.

### Isolation and identification of phenanthrene-degrading endophytic bacterium *Pseudomonas* sp. Ph6

Clover (*Trifolium pratense* L.) grown in PAH-contaminated sites around a petrochemical plant in Nanjing, China, was collected for the isolation of phenanthrene-degrading endophytic bacteria. After collection, plant samples were surface-disinfected to remove epiphytes^8^ and ground in a mortar containing sterile distilled water to obtain plant suspensions. To determine whether the surface disinfection process was successful, plants were pressed onto fresh LB agar plates to detect epiphytic bacteria. A supernatant solution (5 mL) was added to a flask containing 100 mL of PMM. An enrichment culture with phenanthrene-degrading ability was transferred four times to fresh PMM. Pure cultures were obtained by plating a series of dilutions of the enrichment culture solution onto PMM plates^46^.

The morphology of strain Ph6 was determined by conventional methods. The physiological and biochemical characteristics of strain Ph6 were obtained by using the API analytical system. For 16S rRNA gene analysis, the genomic DNA of strain Ph6 was extracted using a DNA Extraction Kit (Tiangen Biotech, Beijing, China), and the 16S rRNA gene was amplified by PCR using the genomic DNA as a template^47^. The 16S rRNA gene sequence was compared with sequences in the GenBank database using the NCBI Blast program (http://blast.ncbi.nlm.nih.gov/Blast.cgi).

### Construction of *gfp*-tagged *Pseudomonas* sp. Ph6 (strain Ph6-*gfp*)

Plasmid pBBRGFP-45 was transformed into *Pseudomonas* sp. Ph6 by triparental conjugation (Table 3). Bacteria containing the plasmid pBBRGFP-45 (Km^r^, 50 mg·L^−1^), bacteria containing plasmid pRK2013 (Km^r^, 50 mg·L^−1^), and strain Ph6 (Cm^r^, 50 mg·L^−1^) were grown separately in LB liquid medium containing appropriate antibiotics overnight at 30°C and 150 r·min^−1^ in a rotary shaker. The bacteria were mixed in equal volumes (v:v:v = 1:1:1), harvested by centrifugation at 10,000 r·min^−1^ for 5 min, and then cultured on a 0.22-μm filter membrane on LB plates overnight. All of the transconjugants that grew on the filter membrane were plated on LB plates containing Cm and Km antibiotics and incubated at 30°C for 1 day to screen for positive transconjugants. The positive transconjugants were purified, and the presence of *gfp* gene was confirmed by PCR. The expression level of *gfp* was examined using fluorescence microscopy under UV light.

### Biodegradation of phenanthrene by Ph6-*gfp* in shake-flask culture

The growth and phenanthrene-degrading ability of strain Ph6-*gfp* were tested. Strain *Pseudomonas* sp. Ph6-*gfp* was grown in LB medium with 50 mg·L^−1^ Cm and 50 mg·L^−1^ Km at 30°C and 150 r·min^−1^. Cells of strain Ph6-*gfp* were harvested by centrifugation, washed three times in MSM, and then adjusted to an approximate suspension of 2.5–5.2 × 10^8^ colony forming units (CFU·mL^−1^). The suspension (1 mL) was used to inoculate 20 mL of fresh PMM media (Containing 0.5‰ yeast extract) at 30°C and 150 r·min^−1^ in a rotary shaker. The population size of strain Ph6-*gfp* was measured with plate counts. Phenanthrene residue in the flask was measured using high-performance liquid chromatography (HPLC; LC-20AT; Shimadzu, Kyoto, Japan) fitted with a 150-mm × 4.6-mm reverse-phase C_18_ column using methanol and water as the mobile phase (v:v = 90:10) at a flow rate of 1 mL·min^−1^. Chromatography was performed at 40°C. Phenanthrene was detected at 245 nm, and the injection volume was 20 μL.

### Greenhouse hydroponic experiments

In the hydroponic experiments, ryegrass was used as the model plant because of its fast growth and well developed root system. Three treatments were designed using batch settings: (1) Contaminated Hoagland medium; (2) Planted ryegrass in contaminated Hoagland medium; and (3) Planted endophyte-inoculated ryegrass in contaminated Hoagland medium. All treatments were conducted in triplicate. A stock methanol solution of phenanthrene was added to the Hoagland medium, and the methanol concentration in Hoagland medium was less than 0.5‰ (v:v). The ryegrass seeds were disinfected with 75% ethanol for 5 min and then placed in a 30°C incubator to germinate for 48 hours.

When ryegrass plants were approximately 10-cm tall, with relatively mature roots, they were transplanted into the Hoagland medium containing phenanthrene (1 mg·L^−1^) contamination for 3 days, with roots submerged in the contaminated Hoagland medium. After the ryegrass plants were contaminated, plant roots were washed with sterile water three times to remove phenanthrene on the root surface. Bacterial inoculum was prepared by resuspending pelleted cells in sterile water to obtain an inoculum density of approximately 9.5 (Log CFU·mL^−1^). Plant roots were thereafter immersed in a suspension of strain Ph6-*gfp* for 6 hours. Then, the plant roots were re-rinsed four times with sterile water, dried on sterile filter paper, and replanted into a 300-mL brown glass bottle containing 250 mL of refresh Hoagland medium with 1 mg·L^−1^ phenanthrene and roots submerged in the contaminated Hoagland medium. For the hydroponic experiments, 10 ryegrass plants were planted in each bottle. Plants with Ph6-*gfp*-free inoculation served as the control. Plant cultivation was carried out in environmental growth chambers set at 25/20°C under a 12-hour light/dark regime for 15 days. Each day, the PAH-free control Hoagland medium was added to the experimental medium to maintain the same initial volume of each treatment. At 3, 6, 9, 12, and 15 days exposure, plant samples were collected from the Hoagland medium and rinsed with sterile water; subsequently, the plant surface was dried with filter paper, and the fresh weight of the plant samples was determined. Fresh plant samples were freeze-dried for 72 hours and reweighed to obtain the dry weight.

### Populations and visualization of strain Ph6-*gfp* within plant tissues

The quantities of strain Ph6-*gfp* in ryegrass roots, shoots, and in the Hoagland medium were determined by counting the CFU on plates. Plant samples were surface-sterilized by sequential immersion in 75% (v:v) ethanol followed by a 0.1% sodium hypochlorite solution. The sterilizing agents were removed from tissues by rinsing three times in sterile water. The surface-sterilized tissues were ground in 5 mL of sterile water. One milliliter of supernatant solution was diluted and grown on LB plates containing two antibiotics (50 mg·L^−1^ Cm and 50 mg·L^−1^ Km), and cell counts of strain Ph6-*gfp* were taken under UV light (green fluorescent colonies).

The localization and distribution of strain Ph6-*gfp* in ryegrass roots and shoots were monitored with fluorescence microscopy. Hand cut, lengthwise sections of surface sterilized root, stem and leaf tissues were fixed on glass slides using 0.25% agarose (m:v) and then observed under a fluorescent microscope. Ph6-*gfp*-free plants were observed under the same conditions and served as controls.

### Analysis of phenanthrene in plant/water/soil

Fresh plant/soil samples were freeze-dried, ground, homogenized, and analyzed for phenanthrene residues by HPLC^49^. Extracts were obtained from a portion of each plant/soil sample by ultrasonication for 30/60 min using an acetone and dichloromethane (DCM) mixture (v:v = 1:1). This acetone/DCM extraction step was repeated three times, and the extracts were combined. The solvents were then evaporated using a rotary evaporator, and the extracts were dissolved in 2 mL of hexane followed by a cleanup procedure through a 2-g silica gel column using 11 mL of 1:1 (v:v) hexane and DCM. The samples were then evaporated and dissolved in methanol, with a final volume of 10 mL. After filtration through 0.22-μm filter units, solutions containing phenanthrene were analyzed by HPLC. Recoveries of phenanthrene investigated by spiking plant samples averaged 103% (n = 5, RSD < 3.3%) for the entire procedure. For water samples, a 10-mL aliquot of a water solution containing phenanthrene was mixed with 10 mL of methanol (HPLC grade), filtered through a 0.22-μm filter unit, and analyzed using HPLC.

### Statistical analysis

All data were processed with Excel 2003 (Microsoft, Redmond, WA). Each data point in the figures and tables represents an average value. Standard deviation in parallel samples is shown in the figures with an error bar. Data were analyzed by ANOVA using SPSS version 11.0 (SPSS, Inc., Chicago, IL) and a confidence limit of 95%.

## Author Contributions

J.L. and Y.Z.G. designed the study. K.S., L.J., Y.J.G. and W.Q.W. conducted the experiments. K.S., J.L. and Y.Z.G. analyzed the data. K.S., J.L. and Y.Z.G. wrote the manuscript.

## Figures and Tables

**Table 1:** Populations of Ph6-*gfp* in ryegrass roots, shoots, and culture solution as determined by counting the CFU on plates. After inoculation for 3–15 days, the quantification of Ph6-*gfp* within plant roots and shoots was performed by counting the CFU on plates containing two antibiotics (50 mg·L^−1^ Cm and 50 mg·L^−1^ Km), and cell counts of strain Ph6-*gfp* were taken under UV light (green fluorescent colonies). In addition, strain Ph6-*gfp* could also be re-isolated and released into culture solution

Time (Days)	Root (Log CFU·g^−1^)	Shoot (Log CFU·g^−1^)	Culture solution (Log CFU·mL^−1^)
3	5.66 ± 0.04	4.07 ± 0.05	4.78 ± 0.07
6	5.79 ± 0.03	4.60 ± 0.04	6.02 ± 0.08
9	5.70 ± 0.02	4.08 ± 0.13	5.96 ± 0.07
12	5.60 ± 0.04	3.94 ± 0.08	5.02 ± 0.03
15	5.51 ± 0.05	3.65 ± 0.10	4.90 ± 0.02

**Table 2:** The accumulation amounts of phenanthrene (*A*) in ryegrass roots and shoots. *A* was estimated according to the following equation: *A* = *C* × *M*, where *C* (mg·kg^−1^) is the concentration of phenanthrene in the root or shoot, and *M* (g·bottle^−1^, on a dry weight basis) is the biomass of ryegrass root or shoot in each bottle. Higher *A* values indicate more phenanthrene present in the plants and higher risks of plant contamination

	No inoculation		Inoculation of Ph6-*gfp*	
Time (Days)	Root (μg·bottle^−1^)	Shoot (μg·bottle^−1^)	Root (μg·bottle^−1^)	Shoot (μg·bottle^−1^)
9	3.64 ± 0.24	0.39 ± 0.07	2.30 ± 0.08	0.27 ± 0.02
12	2.85 ± 0.01	0.55 ± 0.04	2.41 ± 0.07	0.31 ± 0.07
15	0.97 ± 0.02	0.50 ± 0.03	0.87 ± 0.15	0.17 ± 0.04

**Table 3:** Strains and plasmids for triparental conjugation. Bacteria containing the plasmid pBBRGFP-45 (Km^r^, 50 mg·L^−1^), bacteria containing the plasmid pRK2013 (Km^r^, 50 mg·L^−1^), and strain Ph6 (Cm^r^, 50 mg·L^−1^)

Strain or plasmid	Genotype or phenotype	Source or reference
pBBRGFP-45	pBBR-MCS2 vector with 1.4 kb foreign fragment (*gfp*)	Yu^48^
pRK2013	mob^+^, tra^+^, Km^r^, helper plasmid	This laboratory
*Pseudomonas* sp. Ph6	Phenanthrene degradation	This laboratory
